# Correspondence

**Published:** 1860-08

**Authors:** 


MESSRS. EDITORS :—I have just been informed by Dr. T. Morris, of the death from pulmonary consumption of Dr. John Hood, of Greensburg, Ind. Dr. Hood was among the first members of the Indiana State Dental Association, and from the beginning cheerfully gave it his countenance and support. Indeed, he seemed especially attached to it and his profession, being always present at its meetings, even when apparently too feeble to admit of it. Many of the members will recollect how ill he looked when at our last meeting in January; indeed, it was plain to the most casual observer, that the hand of death was upon him, and that he was rapidly leading him away. The 20th of March arrived and John Hood was gone. Dr. Hood had been practicing in Greensburg about nine years, and had accumulated, as I am informed, by a straight forward honorable practice of his profession, a sufficiency to leave his family, consisting of a wife and child, in comparatively easy circumstances. It has been my pleasure at various times, both before and after the death of Dr. H., to meet many of his neighbors, acquaintances, and patients, and I feel that I may safely say that no man in his neighborhood stood higher in point of morality, integrity, or any of the attributes that make the true man, than did he. None can be found who would or could truthfully speak ill of him. As a dentist he was universally regarded by all who knew him, both professional and non-professional, as strictly honest, conscientious, and capable. Always ready to impart information to others, and anxious to acquire more for himself, quite as much for his patients as for his own benefit.

— JOHNSTON.

We knew Dr. Hood, and can fully subscribe to all that is said in the above of him. As a man and a gentleman, we esteemed him highly, as did all who knew him.—ED.

